# Sulforaphane Alleviates Particulate Matter-Induced Oxidative Stress in Human Retinal Pigment Epithelial Cells

**DOI:** 10.3389/fmed.2021.685032

**Published:** 2021-06-17

**Authors:** Hyunchae Sim, Wonhwa Lee, Samyeol Choo, Eui Kyun Park, Moon-Chang Baek, In-Kyu Lee, Dong Ho Park, Jong-Sup Bae

**Affiliations:** ^1^College of Pharmacy, Kyungpook National University, Daegu, South Korea; ^2^Department of Pathology and Regenerative Medicine, School of Dentistry, Kyungpook National University, Daegu, South Korea; ^3^Department of Molecular Medicine, School of Medicine, Kyungpook National University, Daegu, South Korea; ^4^Leading-Edge Research Center for Drug Discovery and Development for Diabetes and Metabolic Disease, Kyungpook National University Hospital, Daegu, South Korea; ^5^Department of Internal Medicine, School of Medicine, Kyungpook National University, Kyungpook National University Hospital, Daegu, South Korea; ^6^Research Institute of Aging and Metabolism, Kyungpook National University, Daegu, South Korea; ^7^Department of Ophthalmology, School of Medicine, Kyungpook National University, Kyungpook National University Hospital, Daegu, South Korea

**Keywords:** age-related macular degeneration, retinal pigment epithelium, oxidative stress, retina, choroid

## Abstract

Age-related macular degeneration (AMD) is a leading cause of blindness in the elderly, and oxidative damage to retinal pigment epithelial (RPE) cells plays a major role in the pathogenesis of AMD. Exposure to high levels of atmospheric particulate matter (PM) with an aerodynamic diameter of <2.5 μm (PM_2.5_) causes respiratory injury, primarily due to oxidative stress. Recently, a large community-based cohort study in the UK reported a positive correlation between PM_2.5_ exposure and AMD. Sulforaphane (SFN), a natural isothiocyanate found in cruciferous vegetables, has known antioxidant effects. However, the protective effects of SNF in the eye, especially in the context of AMD, have not been evaluated. In the present study, we evaluated the effect of SFN against PM_2.5_-induced toxicity in human RPE cells (ARPE-19) and elucidated the molecular mechanism of action. Exposure to PM_2.5_ decreased cell viability in ARPE-19 cells in a time- and dose-dependent manner, potentially due to elevated intracellular reactive oxygen species (ROS). SFN treatment increased ARPE-19 cell viability and decreased PM_2.5_-induced oxidative stress in a dose-dependent manner. PM_2.5_-induced downregulation of serum- and glucocorticoid-inducible kinase 1 (SGK1), a cell survival factor, was recovered by SFN. PM_2.5_ treatment decreased the enzymatic activities of the antioxidant enzymes including superoxide dismutase and catalase, which were restored by SFN treatment. Taken together, these findings suggest that SFN effectively alleviates PM_2.5_-induced oxidative damage in human ARPE-19 cells via its antioxidant effects, and that SFN can potentially be used as a therapeutic agent for AMD, particularly in cases related to PM_2.5_ exposure.

## Introduction

Age-related macular degeneration (AMD) is the most devastating chorioretinal disease, and is a leading cause of blindness in the elderly population (1). The retinal pigment epithelium (RPE) is a monolayer of cells located between the retinal photoreceptors and choroid vascular bed. RPE cells support photoreceptors, which are both postmitotic and highly sensitive to environmental insults, and therefore subject to irreversible degeneration. RPE cells are continuously exposed to reactive oxygen species (ROS) due to light exposure, high retinal oxygen consumption, and abundant polyunsaturated fatty acids and photosensitizers in photoreceptors and the RPE (2). Chronic excessive ROS production and accumulation cause oxidative dysfunction in the RPE, which leads to photoreceptor loss in the advanced form of AMD, geographic atrophy (3).

Increased exposure to particulate matter (PM), especially ultrafine particles with an average aerodynamic diameter of <2.5 μm (PM_2.5_), has been linked to adverse health effects, such as increased risk of cardiovascular and respiratory death (4–6). PM_2.5_ accumulation causes oxidative stress in the body (7), which is considered to be an important molecular mechanism of PM_2.5_-mediated toxicity (8).

**Figure 1:**
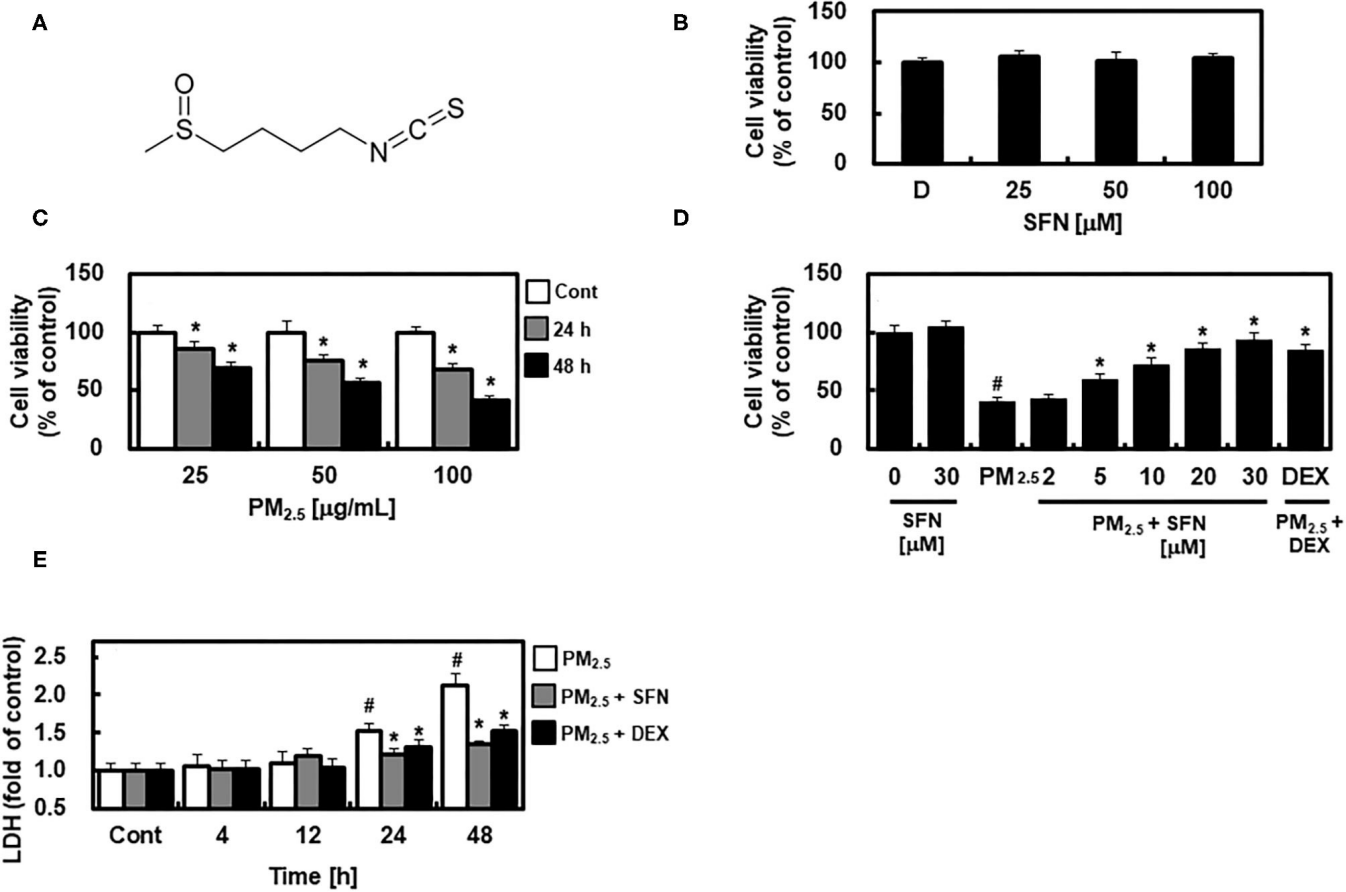
Chemical structure of Sulforaphane (SFN) and effects of SFN on PM_2.5_-induced cell toxicity in ARPE-19 cells. **(A)** Chemical structure of SFN. **(B)** ARPE-19 cells were treated with 0.5% dimethyl sulfoxide as a control indicated as D, and 25, 50, or 100 μM SFN for 24 h, and cell viability was measured using an MTT Assay. **(C)** ARPE-19 cells were treated with 25, 50, or 100 μg/mL PM_2.5_ for 24 or 48 h, and cell viability was measured using an MTT assay. **(D)** ARPE-19 cells were treated with the specified concentrations of SFN or DEX (1 μM) for 6 h after 24 h PM_2.5_ challenge (100 μg/mL). After treatment, cell viability was determined using an MTT assay. **(E)** ARPE-19 cells were treated with SFN (50 μM) or DEX (1 μM) for 6 h after PM_2.5_ challenge (100 μg/mL) for the indicated time periods. Subsequently, LDH levels were determined using an LDH kit. Values represent the mean ± SD of three independent experiments. *p < 0.01 relative to control **(C)** or PM-challenged group **(D,E)**, one-way ANOVA. ^#^*p* < 0.01 relative to control **(D,E)**, one-way ANOVA.

Sulforaphane (SFN) (Figure 1A) is an organosulfur compound found in cruciferous vegetables such as broccoli, Brussels sprouts, and cabbage (9). SFN has attracted particular interest as an indirect antioxidant due to its ability to induce expression of multiple endogenous antioxidant enzymes by activating nuclear factor E2-related factor-2 (Nrf2) (9). Although supplementation of antioxidant agents such as lutein and zeaxanthin has protective effects in AMD (10), the effect of SFN in AMD has not previously been evaluated. In the present study, we aimed to investigate whether SFN could alleviate PM_2.5_-induced oxidative stress in human retinal pigment epithelial cells (ARPE-19), and subsequently to explore the mechanisms underlying the antioxidant effects of SFN in this context.

## Materials and Methods

### Reagents

Diesel PM NIST 1650b (11) was purchased from Sigma-Aldrich (St. Louis, MO, USA), mixed with saline, and sonicated for 30 min to avoid agglomeration of suspended PM_2.5_ particles, as described previously (12). SFN and dexamethasone (DEX), a well-known anti-inflammatory drug (13) used as a positive control, were purchased from Sigma-Aldrich. All other chemicals and reagents were obtained from Sigma-Aldrich unless otherwise stated.

### ARPE-19 Culture and PM_2.5_ Treatment

The human RPE cell line ARPE-19 (ATCC, Manassas, VA, USA, CLR-2302) was maintained in DMEM/F12 medium (Thermo Fisher, Waltham, MA, USA) with 10% FBS and 100 U/mL penicillin−100 μg/mL streptomycin (P/S), and passaged at a ratio of 1:2 to 1:4 using trypsin-EDTA (Thermo Fisher). Cells were grown at 37°C and 5% CO_2_. Cells were grown for 24 h and subsequently treated for 24 h with different concentrations of PM_2.5_ (25, 50, or 100 μg/mL) in the absence or presence of different concentrations of SFN (2, 5, 10, 20, or 30 μM) or DEX (1 μM).

### Cell Viability Assay

A 3-(4,5-dimethylthiazol-2-yl)-2,5-diphenyltetrazolium bromide (MTT) assay was performed to measure cell viability as described previously (12, 14–16). The viability of treated cells was expressed as the percentage of absorbance relative to that of untreated cells, which was assumed to be 100% viability.

### Flow Cytometric Analysis of Apoptosis

Apoptosis was examined using an Annexin V-FITC/PI Apoptosis Detection Kit (BD Biosciences, San Jose, CA, USA) according to the manufacturer's protocol. ARPE-19 cells were grown in a 6-well plate (2 × 10^5^ cells/well) and treated with 100 μg/mL PM_2.5_ for 24 h followed by subsequent treatment with SFN for 6 h. Subsequently, cultured cells in all groups were washed twice with ice-cold PBS, resuspended in 300 μL binding buffer, and stained with 10 μL Annexin V-FITC stock and 10 μL PI in dark conditions for 20 min. Stained cells were immediately analyzed with a FACScan Calibur Flow Cytometer (BD Biosciences), and the number of apoptotic cells was calculated using CellQuest software (Becton–Dickinson, CA, USA). The results were expressed as the percentage of Annexin V-stained cells relative to control, and all experiments were performed in triplicate.

### Western Blot Analysis

For western blot analysis, cells were first rinsed with ice-cold phosphate-buffered saline and treated with lysis buffer comprising 0.5% sodium dodecyl sulfate, 1% NP-40, 1% sodium deoxycholate, 150 mM NaCl, 50 mM Tris-HCl (pH 7.5), and protease inhibitors, as previously described (17). Protein blots were blocked with 5% bovine serum albumin BSA for 2 h and incubated with the following primary antibodies: anti-Bax (1:2000), anti-Bcl2 (1:2000), anti-SGK1 (1:1000), anti-cytochrome c (1:500), and anti-cleaved caspase-3 (1:500) (Cell Signaling Technology, Inc., Danvers, MA, USA). β-actin was used as a loading control. Subsequently, membranes were washed and incubated with horseradish peroxidase-conjugated secondary antibodies (Cell Signaling Technology, 1:5,000). Densitometry analysis was performed using the ImageJ Gel Analysis tool (NIH, Bethesda, MD, USA).

### Lactate Dehydrogenase Assay

To assess the cellular toxicity of PM_2.5_, lactate dehydrogenase (LDH) released from cells after exposure to PM_2.5_ was measured. After 24 h exposure to PM_2.5_ (100 μg/mL), cell-free supernatant aliquots were separated and measured using a commercially available kit (Pointe Scientific, Lincoln Park, MI, USA). All samples were assayed for LDH content in duplicate using a plate reader (Tecan Austria GmbH, Grödig, Austria).

### ROS Measurement

ROS production was determined using 2′, 7′-dichlorodihydrofluorescein diacetate (DCFH-DA). Cells were incubated in a 96-well plate at 2 × 10^5^ cells/well and treated for 4, 12, 24, 48 h with different concentrations of PM_2.5_ (25, 50, or 100 μg/mL). And then, the media were replaced with DCFH-DA (50 μg/mL)-containing media and incubated for 30 min. Intracellular ROS levels were measured by monitoring the fluorescence generated from the oxidation product of DCFH-DA at excitation wavelengths of 485 and 535 nm.

### Evaluation of Oxidative Stress Markers

SOD activity was measured using a SOD assay kit (Fluka). CAT activity was measured using a CAT assay kit (Sigma-Aldrich) based on the decomposition rate of the substrate hydrogen peroxide (H_2_O_2_), which was measured at 240 nm.

### Statistical Analyses

All experiments were performed independently at least three times, and results are expressed as mean ± standard deviation (SD). Statistical significance was analyzed using a one-way analysis of variance (ANOVA) followed by Dunnett's test, with a *p*-value < 0.05 considered statistically significant. SPSS for Windows version 16.0 (SPSS, Chicago, IL, USA) was used to conduct all statistical analyses.

## Results

### Effects of SFN on PM_2.5_-Induced Cell Death and Cytotoxicity

First, we examined the potential cytotoxic effects of SFN in human ARPE-19 cells using an MTT assay. No change in cell viability occurred in cells treated with 0.5% DMSO as a control and different concentrations of SFN ranging from 25 to 100 μM for 24 h (Figure 1B). ARPE-19 cell viability decreased with PM_2.5_ exposure in a dose- and time-dependent manner (Figure 1C), and was recovered by post-treatment with SFN for 6 h (Figure 1D and Supplementary Figure 1). DEX, a well-known anti-inflammatory drug (13, 18), was used as a positive control. Furthermore, cellular LDH release significantly increased after 24 h exposure to PM_2.5_ but decreased after treatment with 30 μM SFN (Figure 1E). These results indicated that the amount of LDH released from cells treated with PM_2.5_ was related to cell viability, and that SFN alleviated PM_2.5_-induced cytotoxicity.

### Effects of SFN on PM_2.5_-Induced Apoptosis

**Figure 2:**
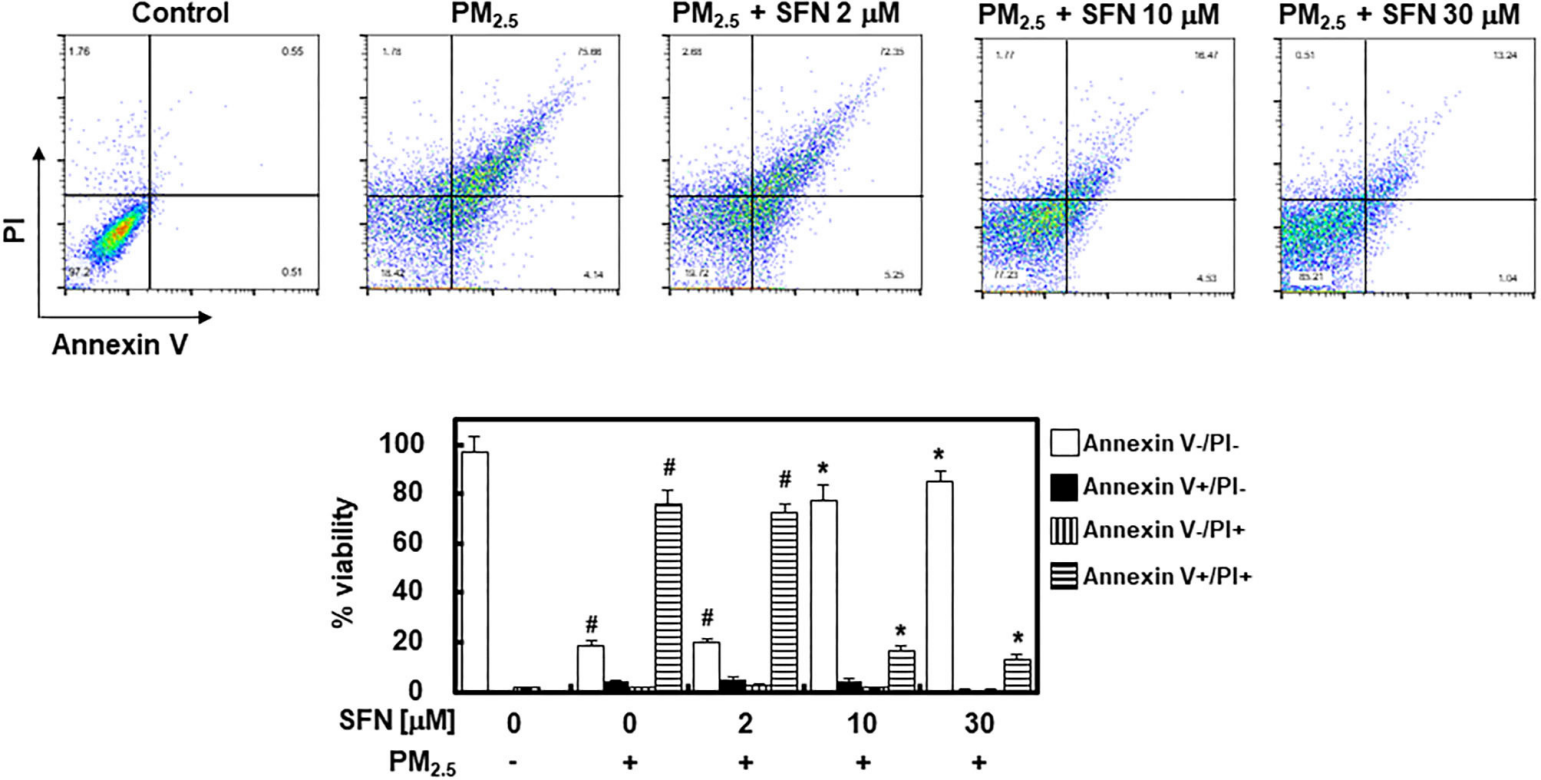
Effects of Sulforaphane (SFN) on PM_2.5_-induced apoptosis in ARPE-19 cells. ARPE-19 cells were treated with PM_2.5_ (100 μg/mL) for 24 h and subsequently treated with the specified concentrations of SFN (0–30 μM) for 6 h. Apoptosis was measured by Annexin V flow cytometry analysis. Values represent the mean ± SD of three independent experiments. *p < 0.01 relative to PM-challenged group, one-way ANOVA. ^#^*p* < 0.01 relative to control, one-way ANOVA.

To further investigate the effect of SFN against PM_2.5_ in ARPE-19 cells, ARPE-19 cell apoptosis was assessed using flow cytometry. Exposure to 100 μg/mL PM_2.5_ for 24 h significantly increased late apoptosis relative to the control group, but post-treatment of ARPE-19 cells with SFN (10 and 30 μM) after PM_2.5_ exposure significantly decreased the PM_2.5_ -induced late apoptosis (Figure 2).

### Effects of SFN on PM_2.5_ Induction of Apoptotic Protein Levels

**Figure 3:**
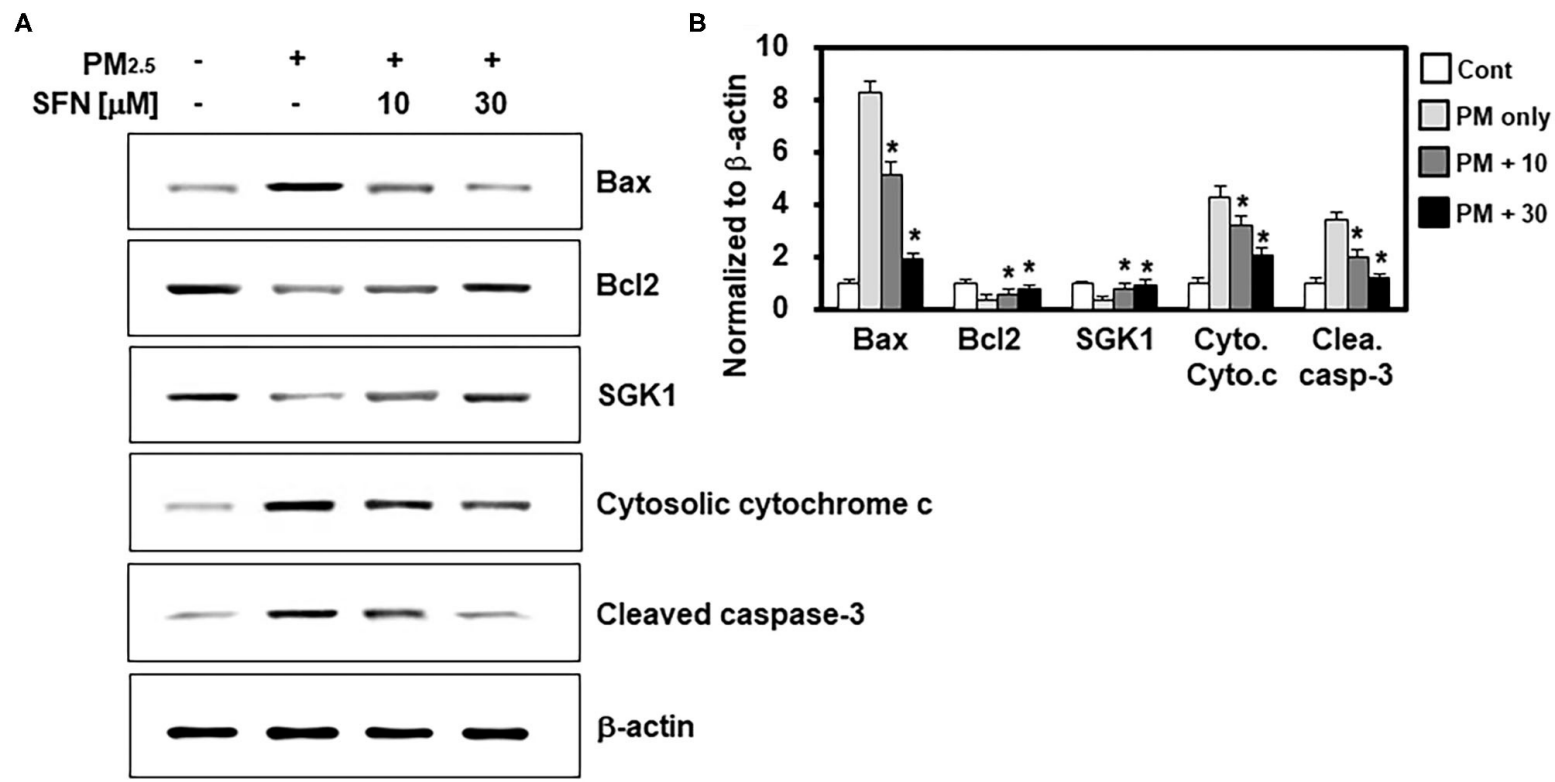
Effects of Sulforaphane (SFN) on PM_2.5_-induced changes in apoptosis-related protein levels. **(A)** ARPE-19 cells were treated with the indicated concentrations of SFN or DEX (1 μM) 24 h after PM_2.5_ challenge (100 μg/mL). Subsequently, western blot analysis was conducted to measure Bax, Bcl-2, SGK1, cytochrome c, and cleaved caspase-3. β-actin was used as a loading control. Representative images from each group are shown (*n* = 3). **(B)** The graphs show the densitometric intensities of each gene normalized to β-actin. *n* = 3 blots. *p < 0.01 relative to the control group, one-way ANOVA.

In light of the effects of SFN against PM_2.5_-induced apoptosis in ARPE-19 cells, we further investigated the effects of SFN on the levels and cleavage of apoptotic proteins, including Bax, Bcl-2, cytochrome c, and caspase-3, by western blotting. Exposure to 100 μg/mL PM_2.5_ (24 h) decreased Bcl-2 and increased Bax, cytochrome c, and cleaved caspase-3 (Figure 3A), which was consistent with the flow cytometry findings. However, post-treatment of ARPE-19 cells with SFN (10 and 30 μM) for 6 h dose-dependently reversed this interaction, as demonstrated by decreased Bax, cytochrome c, and cleaved caspase-3 levels and increased Bcl-2 levels (Figure 3B). Protein levels of SGK1, known as an anti-apoptotic factor (19), were also downregulated by PM_2.5_ treatment and recovered by SFN treatment, suggesting that SGK1 could be relevant to cell survival following PM_2.5_ exposure.

### Effects of SFN on PM_2.5_-Induced ROS Increase

**Figure 4:**
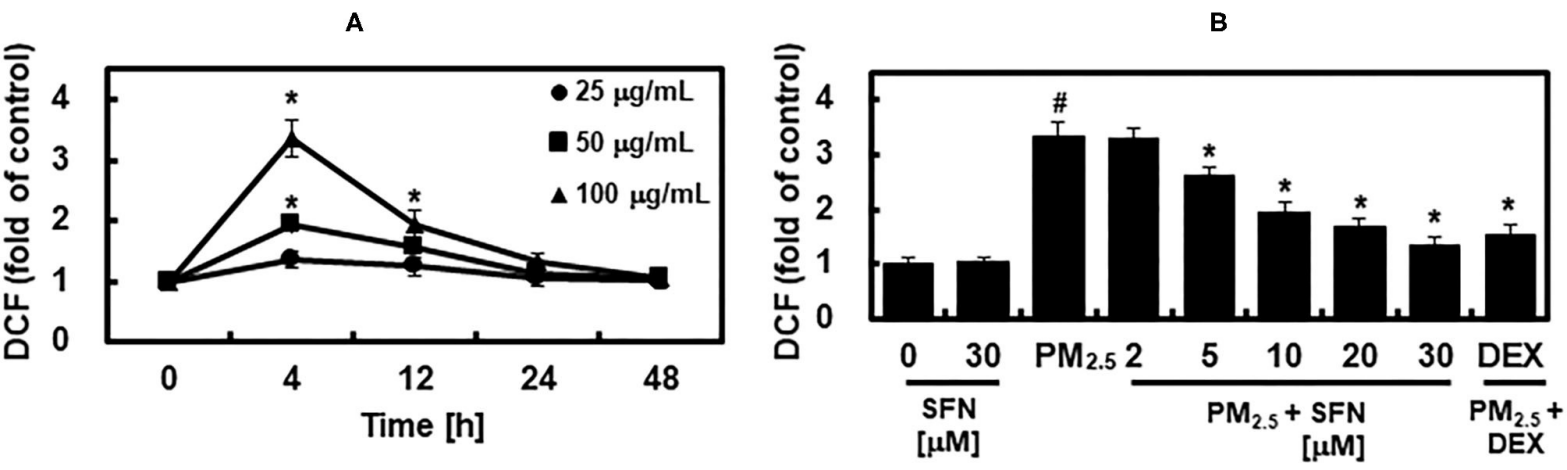
Effects of Sulforaphane (SFN) on PM_2.5_-induced ROS generation. **(A)** ARPE-19 cells were treated for the indicated times with the following concentrations of PM_2.5_: 25 μg/mL (closed circle), 50 μg/mL (closed square), or 100 μg/mL (closed triangle). Intracellular ROS levels were determined using DCFH-DA. DCFH-DA fluorescence values are expressed as the fluorescence ratio (fold) between PM_2.5_-treated cells and untreated control cells. **(B)** ARPE-19 cells were treated with the indicated concentrations of SFN or DEX (1 μM) 24 h after being challenged with PM_2.5_ (100 μg/mL). After treatment, ROS levels were measured. Values represent the mean ± SD of three independent experiments. *p < 0.01 relative to 0 h group **(A)** or PM-challenged group **(B)**, one-way ANOVA. ^#^*p* < 0.01 relative to control **(B)**, one-way ANOVA.

Subsequently, we determined the effects of SFN on PM_2.5_ induction of ROS by measuring DCFH-DA fluorescence intensity in ARPE-19 cells after exposure to 25, 50, or 100 μg/mL PM_2.5_ for 4, 12, 24, or 48 h. PM_25_ exposure increased intracellular ROS levels in a dose-dependent manner (Figure 4A). DCFH-DA fluorescence intensity peaked after 4 h exposure and then dropped to baseline levels after 24 h. Post-treatment with SFN for 6 h after 24 h PM_2.5_ exposure suppressed PM_2.5_-induced ROS in a dose-dependent manner (Figure 4B). DEX decreased ROS levels in PM_2.5_-treated cells (Figure 4B).

### Effects of PM_2.5_ and SFN on Antioxidant Enzyme Activity

**Figure 5:**
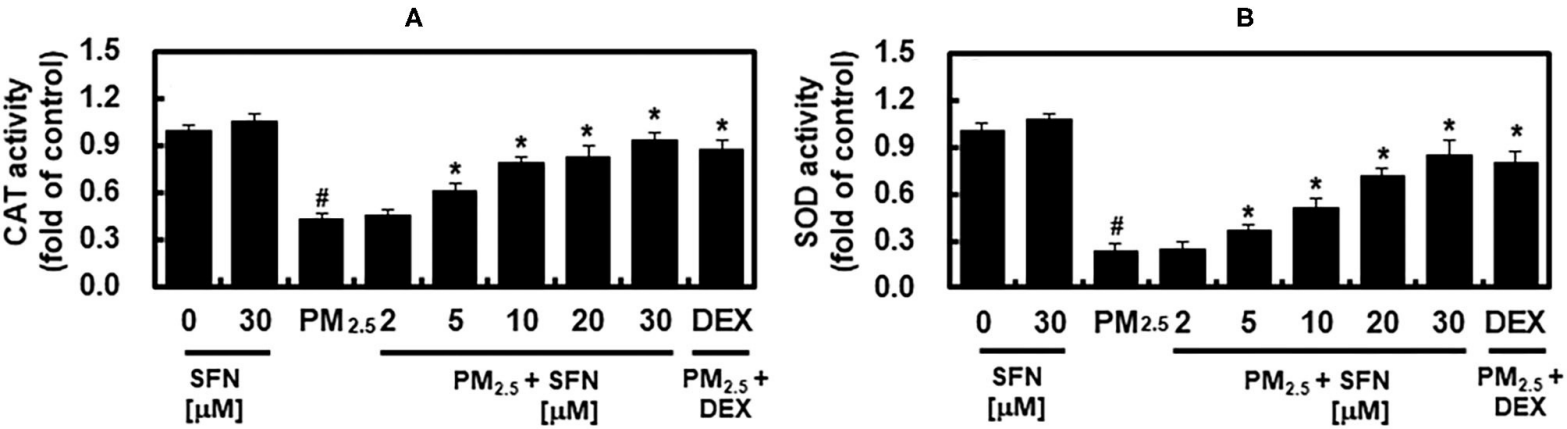
Effects of Sulforaphane (SFN) and PM_2.5_ on antioxidant enzyme activities. ARPE-19 cells were treated with the indicated concentrations of SFN or DEX (1 μM) for 6 after 24 h PM_2.5_ challenge (100 μg/mL). After treatment, the activities of **(A)** catalase (CAT) and **(B)** superoxide dismutase (SOD) were measured. Values represent mean ± SD of three independent experiments. *p < 0.01 relative to PM-challenged group, one-way ANOVA. ^#^*p* < 0.01 relative to control, one-way ANOVA.

The activities of SOD and CAT in ARPE-19 cells were decreased in a dose-dependent manner after 48 h exposure to PM_2.5_, and were recovered by post-treatment with SFN, also in a dose-dependent manner (Figure 5). These results suggested that SFN decreased PM_2.5_-induced oxidative stress by increasing intracellular antioxidant enzyme activity. DEX increased SOD and CAT activities under PM_2.5_ challenge (Figure 5).

## Discussion

A growing body of evidence supports that ROS-induced oxidative stress damages the RPE, which can eventually lead to geographic atrophy and subsequent development of AMD (20, 21). Oxidative stress results primarily from an imbalance between ROS generation and antioxidant defenses, and especially in the context of the RPE, oxidative stress increases with age, leading to photoreceptor impairment and loss (22). Thus, a balanced redox state is crucial for preventing or delaying progression of AMD and vision loss. Consistent with this hypothesis, clinical and basic research studies have demonstrated that daily dietary supplementation of natural antioxidants, such as b-carotenoid, lutein, zeaxanthin, and anthocyanins, inhibits development and progression of AMD (23, 24).

Epidemiological evidence indicates that the greatest health risks posed by environmental PM are associated with ultrafine PM (25). The PM used in the present study was <2.5 μm in diameter, which is known to exert cellular damage in the alveolar regions of the lung (25). Further, a recent study identified that PM_2.5_ promotes epithelial-mesenchymal transition of human RPE, which is mediated by upregulation of TGF-β-dependent nuclear transcription factors (26).

Interestingly, the relationship between air pollution and retinal structure was reported in large community-based cohort studies, collectively referred to as the UK Biobank. Higher concentrations of PM_2.5_ were associated decreased thickness of the ganglion cell-inner plexiform, inner nuclear, and outer plexiform + outer nuclear layers (27). Furthermore, greater exposure to PM_2.5_ was associated with increased incidence of self-reported AMD and decreased thickness of the RPE layer (28).

Despite evidence supporting the association between PM_2.5_ exposure and AMD, PM_2.5_ -mediated oxidative responses and the anti-oxidant effect of SFN, especially in the context of AMD, have not been thoroughly investigated. The purpose of the present study was therefore to examine the potential therapeutic effects of SFN against PM_2.5_-induced RPE cytotoxicity.

The cell viability assay is important in determining the cellular response to toxins, and provides information on cell death, cell survival, and metabolic activities (29). PM_2.5_ is believed to cause genotoxicity and cytotoxicity and suppress cell proliferation (30). The present study demonstrated that PM_2.5_ increased LDH released from ARPE-19 cells, suggesting that PM_25_ exposure decreased cell viability in a time- and dose-dependent manner.

Particles from gasoline engine exhausts filtered by a pore size of 19 μm decrease cell viability in human bronchia epithelium airway cells (31). In addition, exposure to particle suspensions significantly increases LDH levels in rat macrophages (32), which is consistent with our data. In many previous studies, the effect of improving PM_2.5_-caused damage such as pulmonary injury, airway inflammation, and oxidative stress was analyzed in comparison with DEX. Thus, we have scrutinized the efficacy of SFN compared to DEX (33–36). In the present study, SFN reversed PM_2.5_-induced cellular toxicity. Because ROS-triggered apoptosis plays a crucial role in the pathogenesis of AMD (37). Bcl-2 family proteins, including anti-apoptotic proteins, such as Bcl-2 and pro-apoptotic proteins such as Bax, are well-known regulators of apoptosis (38). Prior studies have demonstrated that increases in the Bax/Bcl-2 ratio increase the permeability of the mitochondrial membranes, which results in cytochrome c release and subsequent caspase activation (39, 40). Among activated caspases, cleaved caspase-3 serves as the central executioner in cell death in receptor- or mitochondrial-mediated apoptosis (41). The present study demonstrated that PM_2.5_ exposure increased Bax, cytosolic cytochrome c, and cleaved caspase-3 protein levels and decreased Bcl-2 protein levels. However, post-treatment with SNF after PM_2.5_ exposure effectively reversed these pro-apoptotic changes, including decreased protein levels of Bax, cytosolic cytochrome c, and cleaved caspase-3, and increased Bcl-2 levels. This suggested that elevated intracellular ROS was related to PM_2.5_-induced apoptosis in ARPE-19 cells. Furthermore, previous studies reported that SGK1 promotes cell survival and inhibits cell apoptosis including cardiomyocytes (42). Interestingly, expression of SGK1 was decreased in PM_2.5_-treated human lung alveolar epithelial cells, and overexpression of SGK1 significantly attenuated apoptosis with reduced ROS generation (19). These results were similarly shown in the present study by the SFN treatment. Thus, SFN has a therapeutic effect against PM_2.5_-induced apoptosis in RPE cells by regulating mechanisms upstream of caspase-3, such as antioxidant defense mechanisms.

PM_2.5_ is known to cause oxidative damage (43, 44). Although it is difficult to determine the contribution of PM_2.5_ pollutants to total oxidative burden, many studies have shown that PM_2.5_, metals, carbonaceous materials, and polycyclic aromatic hydrocarbons increase ROS levels (25, 45). PM_2.5_-induced oxidative stress and cytotoxicity are due in part to adsorption of particle transition metals and their oxidation products, which are associated with polycyclic aromatic hydrocarbons (25, 45).

Oxidative stress occurs due to an imbalance between ROS levels and the antioxidant defense mechanisms that quench ROS (46). Antioxidant defense mechanisms, which involve antioxidant enzymes such as SOD, CAT, GSH, and GPx, prevent generation of the most reactive forms of ROS, for example hydroxyl radical, preventing oxidative damage to cellular macromolecules, including DNA, proteins, and lipids (46). SOD catalyzes the dismutation of ${\text{O}}_{2}^{.-}$ to H_2_O_2_, and CAT quenches H_2_O_2_ (47). The present study demonstrated that PM_2.5_ decreased SOD and CAT antioxidant enzyme activities at high concentrations (Figure 5), which is consistent with a prior report that PM impaired the antioxidant enzymatic activities of SOD, GR, CAT, and glutathione-S-transferase in human epithelial cells (48). The results of the present study demonstrated that enzymatic activities of SOD and CAT were decreased by PM exposure, and that these effects were reversed by SFN post-treatment. These results suggest that SFN has antioxidant activity against RPE exposure to PM_2.5_, which was recently identified as a risk factor for AMD (28).

There are several limitations in this study. First, the main limitation is the inability to determine the precise molecular mechanisms of the SFN. Intriguingly, BAK and BAX may not always be required for pro-apoptotic stimuli to promote cytochrome c release and the consequent caspase activation (49). Second, because a wide range of retinal and choroidal pathologies are also involved in AMD such as RPE-Bruch membrane thickening, drusen accumulation, reduced blood flow, photoreceptor degeneration, cofactor accumulation, and inflammatory cytokines and chemokines, our model was not able to explain all of them. Instead, our study focused on the findings that SFN alleviated PM_2.5_-induced RPE cell death in the aspect of oxidative stress suggesting a potential therapeutic for AMD. We will expand our study to focus on other mechanisms such as the complemental pathway (50) and to elucidate the precise molecular mechanism.

Taken together, our findings suggested that PM_2.5_ treatment induced oxidative stress in RPE cells, possibly by elevated intracellular ROS and/or decreasing antioxidant enzyme activity, leading to ARPE-19 cell death. Our findings suggest that PM_2.5_-induced oxidative stress likely exacerbates RPE dysfunction in the context of RPE, and that SFN alleviates PM_2.5_-induced cell death by regulating mechanisms upstream of caspase-3, such as antioxidant defense mechanisms. These findings suggest that SFN is a potential therapeutic for AMD, which is characterized in part by RPE atrophy.

## Data Availability Statement

The data that support the findings of this study are available from the corresponding authors upon reasonable request.

## Ethics Statement

The animal study was reviewed and approved by Animal Care Committee of Kyungpook National University (2019-0104-01).

## Author Contributions

HS, WL, DP, and J-SB: design and conduct of the study, analysis and interpretation of data, writing the manuscript, and critical revision of the manuscript. All authors: collection of data and final approval of the manuscript, contributed to the article, and approved the submitted version.

## Conflict of Interest

The authors declare that the research was conducted in the absence of any commercial or financial relationships that could be construed as a potential conflict of interest.
