# Supplementary material for: Sulforaphane Alleviates Particulate Matter-Induced Oxidative Stress in Human Retinal Pigment Epithelial Cells
Source: Front Med (Lausanne). 2021 Jun 17;8:685032. doi: 10.3389/fmed.2021.685032 (PMC8247919; doi:10.3389/fmed.2021.685032)
Supplement: Supplementary file 1 [file Data_Sheet_1.pdf]

*Supplementary Material*

# Sulforaphane Alleviates Particulate Matter-Induced Oxidative Stress in Human Retinal Pigment Epithelial Cells

**Hyunchae Sim[1§], Wonhwa Lee[1§], Samyeol Choo[1], Eui Kyun Park[2], Moon-Chang Baek[3], In-kyu Lee[4,5,6], Dong Ho Park[4,7*], and Jong-Sup Bae[1**]**

**Correspondence:**
Dong Ho Park
DongHo_Park@knu.ac.kr

Jong-Sup Bae, PhD
baejs@knu.ac.kr

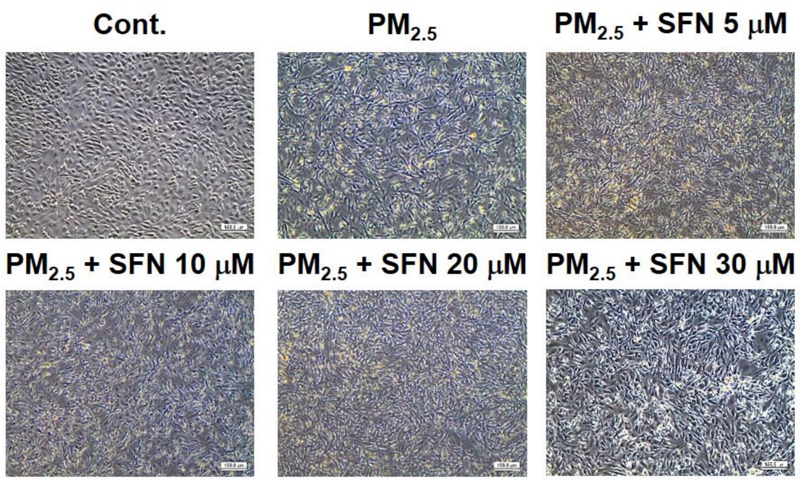


**Supplementary Figure 1.** Representative cell images of the SFN treatment on $PM_{2.5}$-preincubated ARPE-19 cells (Scale bar: 159.8 μm).
